# Supplementary material for: Association between Bone Turnover Markers and Fracture Healing in Long Bone Non-Union: A Systematic Review
Source: J Clin Med. 2024 Apr 17;13(8):2333. doi: 10.3390/jcm13082333 (PMC11051214; doi:10.3390/jcm13082333)
Supplement: Supplementary file 1 [file jcm-13-02333-s001.zip › jcm-2940534-supplementary.pdf]

Table S1. Quality Assessment of the Included Studies.

**References:**	[22]		[23]		[24]		[25]		[26]		[27]		[28]		[29]		[30]		[31]		[32]		[33]		[34]		[35]	
**Reviewers:**	A	B	A	B	A	B	A	B	A	B	A	B	A	B	A	B	A	B	A	B	A	B	A	B	A	B	A	B
**Questionnarie[1]:**																												
Question / objective	2	2	2	2	2	2	2	2	2	2	2	2	2	2	2	2	2	2	2	2	2	2	2	2	2	2	2	2
Study design	2	2	2	2	2	2	2	1	2	2	2	2	2	2	2	2	2	2	2	2	2	2	2	2	1	2	2	2
Subject /comparison group selection (methods)	2	2	2	2	2	2	1	1	2	2	1	1	2	2	2	2	2	2	2	2	2	2	2	2	1	1	2	2
Subject/comparison group characteristics	2	2	2	2	2	2	2	2	2	2	2	2	2	2	2	2	-	-	2	2	2	2	2	2	1	1	2	2
Random allocation description	-	-	1	1	-	-	-	-	-	-	1	1	-	-	-	-	-	-	-	-	-	-	-	-	-	-	-	-
Interventional and blinding of investigators	-	-	1	1	-	-	-	-	-	-	-	-	-	-	-	-	-	-	-	-	-	-	-	-	-	-	-	-
Interventional and blinding of subjects	-	-	2	2	-	-	-	-	-	-	-	-	-	-	-	-	-	-	-	-	-	-	-	-	-	-	-	-
Outcome well defined and robust to measurement	2	1	2	2	2	2	2	1	2	-	1	1	1	2	2	2	2	2	2	2	2	2	2	2	2	2	2	1
Sample size appropriate	1	1	2	2	1	0	1	1	1	0	1	0	1	1	1	1	1	1	1	1	1	2	1	2	1	1	0	0
Analytic methods described and appropriate	2	2	2	2	2	2	1	1	1	1	2	2	2	2	2	2	2	2	2	2	2	2	2	2	1	0	2	2
Estimate of variance for the main results	1	1	2	2	2	2	1	1	2	2	1	1	0	1	2	2	2	2	1	2	1	1	2	1	0	0	0	0
Controlled for confounding	1	1	2	1	1	1	-	1	-	1	1	1	-	1	-	1	2	1	-	-	-	-	-	2	-	1	-	-
Results reported in sufficient detail	2	2	2	1	2	2	2	2	2	2	2	2	2	2	2	2	2	2	2	2	2	2	2	2	2	2	0	1
Conclusions supported by the results	2	2	2	2	2	2	2	2	1	1	1	1	2	2	2	2	2	2	2	2	2	2	1	2	1	2	0	0
**Quality assessment score[2]**	86	82	93	86	91	86	80	68	85	75	71	67	80	86	95	91	95	90	90	95	90	95	90	96	60	64	60	60
**Quality assessment score mean**	84		89		89		74		80		69		83		93		92.5		92.5		92.5		93		62		60	

[1]Kmet et al score [21]: yes=2; partial=1; no=0: not applicable (na): - ; [2] Quality assessment score: (number of ' yes'*2+ number of 'partial'*1) / (28 – (number of "na" x 2)). Legend: A, B: reviewers.

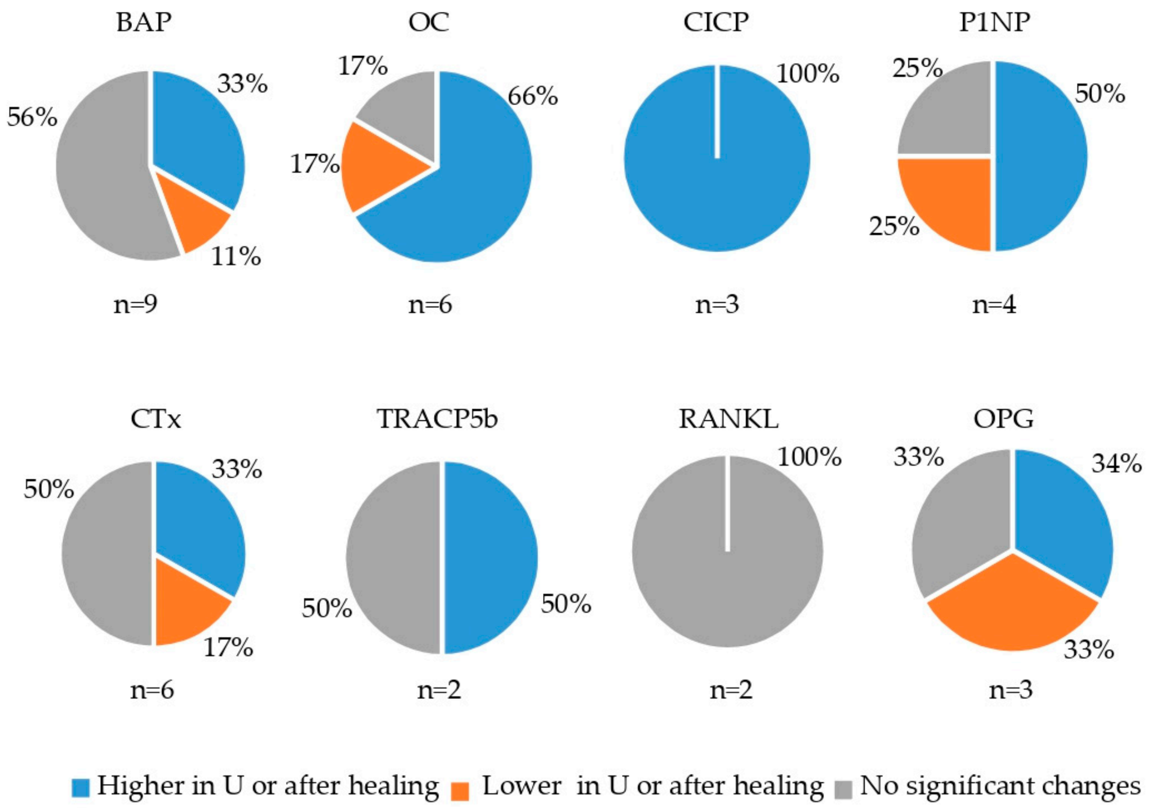

Figure S1. Percentage of studies reporting an increased, decreased or unchanged BTMs level. The increase or decrease refers to changes in respect to control (healed patient or healthy subject) or changes in respect to T0 (surgery) in the same subject/patient. Results were merged independently from endpoint of sampling, that can be different among studies (Table 3). No pie charts is reported for Collagen X and NTX data, as only one study for each BTMs has been found. Legend: BAP: bone alkaline phosphatase. OC: osteocalcin. CICP: C-terminal propeptide and N-terminal (P1NP) are released from procollagen-Type 1. Type-I-Collagen degradation leads to C-terminal telopeptide (CTx) and N-terminal telopeptide (NTX) release. TRACP5b: tartrate-resistant acid phosphatase5b. RANKL: receptor activator of nuclear factor-κB ligand. OPG: osteoprotegerin. n: number of studies.
